# Turkish Adaptation of the Halitosis Associated Life-Quality Test (HALT-TR): Cross-Cultural Adaptation and Initial Psychometric Evaluation in Adults with Self-Perceived Halitosis

**DOI:** 10.3390/healthcare14203413

**Published:** 2026-10-13

**Authors:** Pınar Güvenç, Aslı Silkü Oflioğlu, Deniz Çetin, Hülya Erten Can

**Affiliations:** Department of Restorative Dentistry, Faculty of Dentistry, Dokuz Eylül University, Izmir 35340, Türkiye

**Keywords:** halitosis, self-perceived halitosis, oral health-related quality of life, cross-cultural adaptation, initial psychometric evaluation

## Abstract

**Background/Objectives**: This study translated and culturally adapted the Halitosis Associated Life-Quality Test into Turkish (HALT-TR) and conducted an initial evaluation of its measurement properties in adults with self-perceived halitosis. **Methods**: A total of 200 adults with self-perceived halitosis completed HALT-TR and OHIP-14 before and one week after completion of clinically indicated periodontal and/or restorative treatment. Halitosis was not objectively confirmed, and treatment types were not recorded individually. Internal consistency, dimensional structure, associations with OHIP-14, and standardized treatment-related change were examined. Exploratory PCA and CFA used the same sample. **Results**: All 200 participants had complete responses on both instruments at both assessments. Cronbach’s α was 0.918. PCA identified three components, but CFA showed inadequate fit (RMSEA = 0.18; CFI = 0.88). The HALT-TR–OHIP-14 correlation was absent at baseline (ρ = −0.080; *p* = 0.259), weak after treatment (ρ = 0.266; *p* < 0.001), and absent for individual changes (ρ = −0.091; *p* = 0.200). Mean HALT-TR scores decreased from 38.33 ± 15.42 to 9.58 ± 14.13 (SRM = 1.35). At follow-up, 40.5% had a HALT-TR total score of zero. **Conclusions**: HALT-TR showed high internal consistency and substantial standardized short-term treatment-related change. Structural validity and convergent validity remain insufficiently established. The total score is preliminary, and routine clinical use requires further evaluation.

## 1. Introduction

Halitosis, defined as an unpleasant odor originating from the oral cavity or exhaled air, is a common condition that can adversely affect individuals’ quality of life. Its impact extends beyond clinical manifestations and may affect various aspects of life, including social interactions, psychological well-being, and self-confidence [1,2]. Evidence from systematic reviews and meta-analyses has demonstrated a significant association between halitosis and impaired oral health-related quality of life (OHRQoL) [1]. However, individuals’ perceptions of their own breath odor do not always correspond with objective measures of halitosis, and self-perceived halitosis may have a substantial impact on quality of life [3,4]. Therefore, a comprehensive assessment of halitosis should consider not only objective clinical measures but also the impact of the condition as perceived by the individual.

Previous studies evaluating the impact of halitosis on quality of life have used generic OHRQoL measures, such as the Oral Health Impact Profile (OHIP) and Oral Impacts on Daily Performance (OIDP), as well as generic health-related quality-of-life instruments, such as the Short Form-36 (SF-36) [3]. However, these generic measures may lack the specificity required to fully capture the condition-specific social and psychosocial impact of halitosis on quality of life [3,5]. Assessing the specific effects of halitosis on interpersonal communication, social interactions, self-confidence, and psychological well-being, as well as monitoring patient-perceived changes following treatment, therefore requires a halitosis-specific patient-reported outcome measure (PROM) [5,6].

To address this need, Kizhner et al. developed the Halitosis Associated Life-Quality Test (HALT) [7]. HALT is a 20-item, halitosis-specific quality-of-life instrument designed to assess the physical, emotional, and functional limitations associated with halitosis, as well as its personal and social impact. Each item is scored from 0 to 5, yielding a total score ranging from 0 to 100, with higher scores indicating a greater negative impact of halitosis on quality of life. In the original development study, HALT demonstrated high internal consistency, a strong association with organoleptic assessments, and the ability to detect changes following treatment [7].

HALT has subsequently been adapted into different languages and cultural contexts, and its psychometric properties have been evaluated in various populations. The Chinese version demonstrated high internal consistency and test–retest reliability, with factor analysis yielding a four-factor structure [5]. In contrast, factor analysis of the Brazilian adult version supported a unidimensional structure, while the instrument demonstrated high internal consistency and test–retest reliability [6]. The Romanian version also demonstrated high internal consistency and test–retest reliability, with factor analysis yielding a four-factor structure [8]. In the Thai validation study, exploratory factor analysis identified a four-factor structure, which was subsequently evaluated using confirmatory factor analysis; however, two items had to be removed from the multidimensional model because of low standardized factor loadings [3]. These findings indicate that although HALT generally demonstrates strong psychometric properties across different languages and cultures, findings regarding its factor structure are not entirely consistent across populations [3,5,6,8]. Therefore, adaptation of HALT to a new language and cultural context should extend beyond linguistic translation to include cultural adaptation and re-evaluation of its psychometric properties in the target population [3,5].

Contemporary guidance from the COnsensus-based Standards for the selection of health Measurement INstruments (COSMIN) emphasizes that the measurement properties of a PROM should be evaluated separately and interpreted in relation to its intended construct, target population, and context of use. Different language versions may have different measurement properties; therefore, evidence obtained for one version should not automatically be assumed to apply to another [9]. Updated reporting recommendations also emphasize transparent reporting of studies evaluating PROM measurement properties [10]. These considerations support an evaluation of HALT-TR that distinguishes internal consistency from structural validity and examines longitudinal score changes without assuming that all aspects of measurement quality have been established.

The present study addresses the need for a culturally adapted assessment of halitosis-related quality of life in Turkish-speaking adults. Beyond linguistic adaptation, its contribution lies in jointly examining item-level performance, dimensional structure, associations with a generic oral health-related quality-of-life measure, and short-term changes following clinically indicated dental treatment. This approach provides an opportunity to assess whether the translated items function coherently in the target population and whether changes in halitosis-specific scores correspond to changes in broader oral health impacts. Accordingly, this study aimed to translate and culturally adapt HALT into Turkish and evaluate the internal consistency and dimensional structure of HALT-TR, its associations with OHIP-14, and changes in its scores from baseline to one week after treatment completion. These analyses were intended to provide initial evidence for its use in Turkish-speaking adults and identify measurement properties requiring further validation.

## 2. Materials and Methods

### 2.1. Study Design and Participants

This methodological study cross-culturally adapted the Halitosis Associated Life-Quality Test (HALT) into Turkish and conducted an initial psychometric evaluation of HALT-TR in adults with self-perceived halitosis. Adult patients presenting with a complaint of halitosis to the Dokuz Eylül University Oral and Dental Health Application and Research Center were recruited. No objective confirmation of oral malodor was required.

Individuals were eligible if they were aged 18 years or older, reported a complaint of halitosis, were literate in Turkish, were able to understand and complete the questionnaire independently, and provided written informed consent. Individuals who were unable to read Turkish, had cognitive impairment that precluded independent questionnaire completion, declined participation or informed consent, or had an active psychiatric disorder were excluded.

Given the 20-item structure of HALT, the target sample size was initially based on the commonly used recommendation of approximately 5–10 participants per item, and 200 participants were included. However, factor-analytic sample adequacy depends on features such as communalities, factor overdetermination, and model complexity rather than on a universal participant-to-item ratio [11]. Accordingly, this sample size does not justify treating exploratory and confirmatory analyses performed in the same participants as independent validation.

### 2.2. Ethical Approval and Informed Consent

The study was conducted in accordance with the research protocol approved by the Dokuz Eylül University Non-Interventional Research Ethics Committee (decision No. 2026/22-03; 15 June 2026). Before enrollment, all participants were informed about the purpose and scope of the study and provided written informed consent. Participant confidentiality was protected, and study data were recorded using anonymized codes and used solely for scientific research purposes.

### 2.3. Halitosis Associated Life-Quality Test (HALT)

HALT is a 20-item, condition-specific patient-reported outcome measure developed to assess the impact of halitosis on daily life and quality of life. Each item is scored from 0 to 5, yielding a total score ranging from 0 to 100. No items are reverse scored. Higher scores indicate a greater negative impact of halitosis on quality of life.

Because previous cross-cultural adaptations of HALT have reported heterogeneous factor structures, no predefined subscale scoring system was imposed on HALT-TR. The dimensional structure of the Turkish version was examined empirically, while the original 20-item total score was retained as the principal scoring approach.

### 2.4. Translation and Cultural Adaptation of HALT-TR

The cross-cultural adaptation process comprised forward translation, review for semantic and conceptual equivalence, back-translation, and pilot testing. The original English HALT was translated into Turkish by a native English speaker working as an English-language teacher in Türkiye. The researchers and translator reviewed the Turkish version for semantic equivalence, conceptual consistency, and cultural appropriateness.

The Turkish version was independently back-translated into English by a native Turkish-speaking professor of English philology who had not seen the original English items. The back-translation was compared with the original HALT to identify possible differences in meaning; discrepancies were reviewed by the research team.

The preliminary Turkish version was pilot-tested in 30 individuals, who provided favorable feedback on the questionnaire. No changes to the items or response options were considered necessary after pilot testing, and this version was retained as the final HALT-TR.

### 2.5. Oral Health Impact Profile-14 (OHIP-14)

The validated Turkish version of the Oral Health Impact Profile-14 (OHIP-14) was administered alongside HALT-TR to assess oral health-related quality of life [12,13]. OHIP-14 comprises 14 items scored from 0 to 4, producing a total score of 0–56; higher scores indicate a greater negative impact of oral health problems on quality of life. Although OHIP-14 encompasses seven conceptual domains, only the total score was used in the present study because the primary objective was the validation of HALT-TR. OHIP-14 was not treated as a gold standard for HALT-TR, but as a related patient-reported outcome measure for evaluating the association between halitosis-specific and general oral health-related quality of life.

### 2.6. Data Collection and Pre- and Post-Treatment Assessments

Sociodemographic characteristics, including age, sex, educational level, and marital status, were recorded at baseline. Participants then completed HALT-TR and OHIP-14 in an appropriate setting that ensured privacy. Each participant was assigned a unique study code independent of personal identifiers.

Following baseline assessment, participants received periodontal and/or restorative treatment according to their clinical needs. HALT-TR and OHIP-14 were re-administered to the same participants one week after completion of the planned dental treatment. Individual treatment categories and pretreatment clinical findings were not available in the questionnaire datasets and could not be summarized by participant.

Individual change scores were calculated as the pretreatment score minus the post-treatment score (ΔHALT = HALTpre − HALTpost; ΔOHIP-14 = OHIP-14pre − OHIP-14post). Accordingly, positive change scores represented a reduction in questionnaire scores and therefore improvement after treatment.

### 2.7. Psychometric Evaluation of HALT-TR

#### 2.7.1. Internal Consistency and Item Analysis

Internal consistency of HALT-TR was assessed using Cronbach’s alpha (α). Corrected item–total correlations were calculated to evaluate the relationship of each item with the remainder of the scale. Cronbach’s alpha, if an item was deleted, was also examined to determine the effect of removing each item on overall internal consistency.

#### 2.7.2. Construct Validity and Factor Analysis

Sampling adequacy was assessed by the Kaiser–Meyer–Olkin measure and Bartlett’s test. The original analysis used principal component analysis (PCA) with Varimax rotation and the eigenvalue >1 rule. In a post-review sensitivity analysis using the complete baseline item data (*n* = 200), ordinal pairwise polychoric correlations were estimated by maximizing bivariate ordinal-normal likelihoods, with thresholds determined by marginal response frequencies. The number of dimensions was assessed using Horn’s parallel-analysis principle [14] by comparing observed eigenvalues with the 95th percentiles from 100 independently permuted ordinal datasets that preserved each item’s response frequencies (random seed 4571899). A common-factor model was extracted from the polychoric correlation matrix using unweighted least squares to minimize off-diagonal residuals, followed by Varimax rotation. The rotated loadings were inspected for interpretability and cross-loadings. This post-review exploratory analysis does not provide independent validation of a factor structure.

The original PCA-derived structure was evaluated using confirmatory factor analysis (CFA) in the same 200 participants; this is a nonindependent appraisal, not an external confirmation. Model fit was assessed using the chi-square to degrees-of-freedom ratio (χ^2^/df), root mean square error of approximation (RMSEA), standardized root mean square residual (SRMR), comparative fit index (CFI), and goodness-of-fit index (GFI).

### 2.8. Association Between HALT-TR and OHIP-14

Associations between HALT-TR and OHIP-14 total scores were examined separately at pretreatment and post-treatment assessments. The association between individual change scores (ΔHALT and ΔOHIP-14) was also evaluated. Spearman’s rank correlation coefficient (ρ) was used for these analyses. OHIP-14 was considered a related construct rather than a reference or gold-standard measure.

### 2.9. Standardized Treatment-Related Change

Pretreatment and post-treatment total scores were compared to evaluate treatment-related change in HALT-TR and OHIP-14. The magnitude of within-person change was summarized using the standardized response mean (SRM), calculated as the mean change score divided by the standard deviation of the change scores. Because change scores were defined as pretreatment minus post-treatment values, positive SRM values indicated improvement. The SRM was used to describe standardized change and was not interpreted as a threshold for clinically meaningful improvement.

### 2.10. Secondary Analyses of Sociodemographic Variables

Age, sex, educational level, and marital status were used to characterize the sample. Associations of age with HALT-TR scores and change were assessed by Spearman’s rank correlation. Exploratory comparisons of repeated HALT-TR and OHIP-14 total scores by sex, educational level, and marital status used the Brunner–Langer nonparametric F1-LD-F1 model. Group, time, and group × time effects were assessed with ANOVA-type statistics (ATS); these effects describe score patterns and do not establish differential measurement properties.

### 2.11. Statistical Analysis

Continuous variables were summarized as mean ± standard deviation (SD) and/or median with interquartile range (IQR), as appropriate, and categorical variables as number (n) and percentage (%). All statistical tests were two-sided, and *p* < 0.05 was considered statistically significant.

Internal consistency was evaluated using Cronbach’s alpha, corrected item–total correlations, and Cronbach’s alpha if item deleted. Factorability was assessed using the KMO measure and Bartlett’s test of sphericity; PCA with Varimax rotation and CFA were used to investigate construct validity. Associations between HALT-TR and OHIP-14 scores were examined using Spearman’s rank correlation.

For longitudinal comparisons across sociodemographic groups, a nonparametric repeated-measures model based on the Brunner–Langer approach (F1-LD-F1) was used. Group effects, time effects, and group × time interactions were evaluated using ANOVA-type statistics (ATS). Standardized pre–post change was summarized using the SRM. The original longitudinal group analyses were conducted with nparLD version 2.2 in R version 4.4.1, as identified in the analysis reports. Other original analyses were reported as performed in IBM SPSS Statistics version 30.0 (IBM Corp., Armonk, NY, USA).

## 3. Results

### 3.1. Participant Characteristics

The four questionnaire datasets contained complete pretreatment and one-week post-treatment HALT-TR and OHIP-14 responses for the same 200 participant identifiers, without missing item responses. The number screened and the distribution of specific dental treatments were not available in these datasets. A total of 200 participants were included. The mean age was 40.20 ± 15.52 years (range, 19–87 years), and the median age was 37 years. The available dataset comprised two sex categories (*n* = 129 [64.5%] and *n* = 71 [35.5%]), three educational-level categories (*n* = 17 [8.5%], *n* = 118 [59.0%], and *n* = 65 [32.5%]), and four marital-status categories (*n* = 70 [35.0%], *n* = 9 [4.5%], *n* = 4 [2.0%], and *n* = 117 [58.5%]) (Table 1).

### 3.2. Internal Consistency and Item Analysis of HALT-TR

HALT-TR demonstrated high internal consistency at the pretreatment assessment, with a Cronbach’s alpha of 0.918 for the 20-item scale (participant-bootstrap 95% CI, 0.896–0.934; 3000 resamples). Corrected item–total correlations ranged from −0.004 to 0.803 (Table 2). The first three items showed lower corrected item–total correlations (−0.004, 0.088, and 0.242, respectively) than the remaining items, for which correlations ranged from 0.371 to 0.803. The highest corrected item–total correlation was observed for item 10 (r = 0.803).

Cronbach’s alpha, if an item was deleted, ranged from 0.909 to 0.925. Removal of items 1, 2, or 3 increased the overall alpha only modestly, to 0.925, 0.922, and 0.921, respectively. The original 20-item composition was retained for subsequent analyses.

### 3.3. Construct Validity and Factor Structure of HALT-TR

The KMO measure was 0.904, and Bartlett’s test of sphericity was statistically significant (*p* < 0.001). The original PCA with Varimax rotation identified three components with eigenvalues greater than 1, explaining 66.47% of total variance. In the ordinal sensitivity analysis, the first four eigenvalues of the polychoric correlation matrix were 9.346, 3.020, 1.764, and 1.051, compared with parallel-analysis 95th percentiles of 1.797, 1.671, 1.529, and 1.458, respectively. Thus, three eigenvalues exceeded their comparison values. Three-factor common-factor extraction from the polychoric matrix nevertheless yielded a weakly defined third factor: Items 1–3 loaded 0.317, 0.988, and 0.518, respectively, on that factor, while several other items loaded on more than one factor (Table 3A). These findings do not establish interpretable and stable HALT-TR subscales.

The original nonindependent CFA of the three-component structure yielded χ^2^/df = 7.22, RMSEA = 0.18, SRMR = 0.13, CFI = 0.88, and GFI = 0.62. Taken together, these indices indicated inadequate model fit. These findings did not support the proposed three-component structure or separate subscale scoring. The original 20-item total score was retained for subsequent analyses; however, the structural validity of the total score remains unresolved (Table 3B).

### 3.4. Association Between HALT-TR and OHIP-14

At pretreatment assessment, HALT-TR and OHIP-14 total scores were not significantly correlated (Spearman’s ρ = −0.080, *p* = 0.259; bootstrap 95% CI, −0.218 to 0.059). The post-treatment correlation was weak (ρ = 0.266, *p* < 0.001; 95% CI, 0.124 to 0.397). Individual change scores were not significantly correlated (ρ = −0.091, *p* = 0.200; 95% CI, −0.231 to 0.048) (Table 4). Confidence intervals were obtained using 3000 participant-level bootstrap resamples with a fixed random seed.

### 3.5. Standardized Treatment-Related Change

Mean HALT-TR total scores decreased from 38.33 ± 15.42 at baseline to 9.58 ± 14.13 at follow-up. The mean change was 28.76 ± 21.32 points (95% CI, 25.78–31.73), and the median change was 33 (Q1–Q3, 18.5–42). The paired Wilcoxon signed-rank statistic was W = 803 (two-sided *p* < 0.0001). The SRM was 1.35, describing standardized treatment-related change rather than established responsiveness.

Mean OHIP-14 total scores decreased from 26.06 ± 9.56 at baseline to 5.98 ± 6.84 at follow-up. The mean change was 20.08 ± 8.13 points (95% CI, 18.95–21.21), and the median change was 20 (Q1–Q3, 14–27). The paired Wilcoxon signed-rank statistic was W = 4 (two-sided *p* < 0.0001); the SRM was 2.47 (Table 5). The HALT-TR total score was zero in 1/200 (0.5%) participants at baseline and 81/200 (40.5%) at follow-up; no participant scored the maximum of 100. OHIP-14 scores were zero in 1/200 (0.5%) at baseline and 74/200 (37%) at follow-up, while 1/200 (0.5%) scored the maximum of 56 at baseline. This pronounced follow-up floor effect limits discrimination between participants reporting little or no remaining impact.

### 3.6. Secondary Analyses of Sociodemographic Variables

In the exploratory Brunner–Langer analyses, HALT-TR scores showed a time effect (ATS = 313.229, *p* < 0.0001), with no sex effect (ATS = 0.397, *p* = 0.529) or sex × time interaction (ATS = 0.002, *p* = 0.963). OHIP-14 also showed a time effect (ATS = 538.963, *p* < 0.0001), but neither a sex effect (ATS = 1.370, *p* = 0.242) nor a sex × time interaction (ATS = 0.973, *p* = 0.324).

By educational level, HALT-TR showed a time effect (ATS = 216.242, *p* < 0.0001), without an educational-level effect (ATS = 0.570, *p* = 0.489) or interaction (ATS = 0.690, *p* = 0.456). OHIP-14 showed an educational-level effect (ATS = 6.415, *p* = 0.0039), a time effect (ATS = 672.781, *p* < 0.0001), and an interaction (ATS = 25.322, *p* < 0.0001). The educational-level categories contained 17, 118, and 65 participants, respectively.

By marital status, HALT-TR showed no overall marital-status effect (ATS = 1.112, *p* = 0.309), but did show a time effect (ATS = 378.176, *p* < 0.0001) and an interaction (ATS = 7.664, *p* = 0.0006). OHIP-14 showed a time effect (ATS = 282.344, *p* < 0.0001) and an interaction (ATS = 3.855, *p* = 0.0202), while its overall marital-status effect was not statistically significant (ATS = 2.976, *p* = 0.0572). Marital-status groups comprised 70, 9, 4, and 117 participants; subgroup results with very small cell sizes should be interpreted cautiously.

Age showed a weak positive correlation with pretreatment HALT-TR score (ρ = 0.174, *p* = 0.014), a weak negative correlation with post-treatment HALT-TR score (ρ = −0.277, *p* < 0.001), and a weak positive correlation with ΔHALT (ρ = 0.289, *p* < 0.001).

## 4. Discussion

The present study translated and culturally adapted HALT into Turkish and examined its measurement performance in adults with self-perceived halitosis. HALT-TR showed high internal consistency and a large standardized decrease in scores one week after completion of dental treatment. However, CFA did not support the three-component structure identified by PCA, and associations with OHIP-14 were limited. The study therefore contributes initial evidence on item performance and short-term score changes in a Turkish-speaking clinical population, while identifying structural validity, temporal stability, and interpretation of individual change as priorities for further investigation.

The internal consistency of HALT-TR was high, with a Cronbach’s alpha of 0.918. This finding is consistent with previous cross-cultural validations of HALT, which have generally reported high internal consistency. The Chinese version demonstrated a Cronbach’s alpha of 0.95 [5], while the Brazilian version reported an alpha of 0.96 [6]. Similarly, high internal consistency was reported for the Romanian adaptation [8], and the more recent Thai validation reported Cronbach’s alpha values of 0.940 and 0.943 across two assessment phases [3]. However, internal consistency should be interpreted in conjunction with structural validity rather than as independent confirmation of the instrument’s validity. The COSMIN framework treats these as distinct measurement properties [15,16], and its updated guidance emphasizes their interdependence [9]. In the present study, Cronbach’s α of 0.918 indicates substantial interrelatedness among the items, but the inadequate CFA fit prevents this coefficient from being interpreted as confirmation of a coherent unidimensional total score. Moreover, high internal consistency does not establish temporal stability, which was not assessed in this study. The findings therefore provide encouraging but incomplete evidence regarding the measurement performance of HALT-TR.

Despite the high overall internal consistency, the first three HALT-TR items showed comparatively weak corrected item–total correlations. This finding deserves consideration because these items primarily address conditions or symptoms potentially associated with oral malodor, whereas many of the subsequent HALT items more directly address the emotional, social, and behavioral consequences of halitosis [7]. A similar item-level issue has been reported in the recent Thai validation, in which removal of Items 1, 3, and 7 resulted in small increases in Cronbach’s alpha, and Item 1 subsequently showed inadequate loading in the multidimensional CFA model [3]. These observations suggest that some of the early HALT items may behave differently from items that more directly represent quality-of-life consequences and that their psychometric performance may also vary across cultural settings. Nevertheless, removal of Items 1–3 in the present study produced only modest increases in Cronbach’s alpha. Accordingly, these items were retained to preserve the content and comparability of the original 20-item HALT rather than creating a modified Turkish version on the basis of a single validation sample. Item-level differences across translated versions of a PROM may also arise from linguistic or cultural variation in how particular symptoms, experiences, and social consequences are understood. Cross-cultural adaptation therefore requires preservation of conceptual rather than merely literal equivalence, with attention to the relevance and comprehensibility of individual items in the target culture [17]. The retention of Items 1–3 in HALT-TR is consistent with this conservative approach, because their removal on the basis of a single dataset could alter the content represented by the original instrument and reduce comparability with other HALT adaptations. In particular, Item 1 had an almost zero corrected item–total correlation, so the issue is not adequately explained by a small increase in alpha after deletion. The content of these items, response distributions, possible differences in Turkish interpretation, and their relevance to the intended quality-of-life construct should be examined through cognitive interviews and replication before any item is revised or removed.

The factor-analytic findings provide further evidence that the dimensional structure of HALT may not be entirely stable across cultural adaptations. In the present study, the KMO value of 0.904 and significant Bartlett’s test indicated that the data were suitable for factor analysis. PCA identified three components accounting for 66.47% of the total variance; however, several items showed cross-loadings, and CFA of the resulting structure demonstrated inadequate model fit. This differs from the Chinese validation, in which four factors explaining 85.18% of the variance were identified [5]. The 2024 Thai validation identified four factors, but Items 1 and 7 were excluded from the multidimensional CFA model because of poor standardized loadings [3]. This distinction matters when comparing its findings with the present study, in which all 20 items were retained. Differences in model fit cannot therefore be attributed solely to language or culture, because the evaluated item sets also differed. The Thai findings highlight the need to examine item-level performance alongside overall model fit, without assuming that the same item modifications would be appropriate for HALT-TR. In contrast, the Brazilian validation supported a unidimensional solution [6], further demonstrating that the latent organization of HALT has not been reproduced consistently across language versions. The post-review common-factor analysis of ordinal responses and parallel analysis also suggested three dimensions, but the third factor was predominantly defined by the first three symptom-related items, with a loading close to unity for Item 2 and several cross-loadings elsewhere. Factor retention alone does not establish a clinically interpretable scoring structure. The Varimax rotation imposed zero factor correlations by design; correlations among underlying constructs cannot be inferred from this orthogonal solution.

Differences between language versions may reflect variation in item interpretation, sample characteristics, or analytical methods; the present findings cannot determine the relative contribution of these explanations. Contemporary COSMIN guidance emphasizes that measurement properties should be evaluated for each language version and scoring structure rather than inferred from findings obtained in other populations [9]. In the present sample, the inadequate fit of the PCA-derived three-component model indicates that this structure was not supported. However, rejection of this model does not establish that HALT-TR is unidimensional or validate the use of a single total score. The original 20-item total score was retained in the analyses to preserve the original scoring approach, but its interpretation remains provisional pending further structural validation. Separate subscale scores cannot currently be recommended. Independent evaluation of the dimensional structure is needed before either total-score or subscale-based interpretations can be considered well established.

The associations between HALT-TR and OHIP-14 provide limited support for convergent validity. The baseline correlation was small and not statistically significant (ρ = −0.080, *p* = 0.259), whereas the post-treatment correlation was statistically significant but weak (ρ = 0.266, *p* < 0.001). Individual changes in the two instruments were also not significantly correlated (ρ = −0.091, *p* = 0.200). HALT assesses halitosis-specific quality-of-life impacts, whereas OHIP-14 measures broader oral health impacts [7,13]; differences in construct coverage may therefore partly explain these findings. Nevertheless, this explanation remains a hypothesis rather than evidence that HALT-TR captures a distinct construct successfully. In particular, the weak post-treatment association should not be overstated on the basis of statistical significance alone. These results indicate that the relationship between HALT-TR and broader oral health-related quality of life requires further investigation.

This distinction is also consistent with broader evidence showing that the relationship between halitosis and quality of life is complex and depends partly on the way halitosis and OHRQoL are assessed. A systematic review and meta-analysis involving 2692 participants found that halitosis was associated with impaired OHRQoL overall, while also identifying considerable heterogeneity across the included studies [1]. In addition, a population-based study of 1022 Brazilian adults found that self-reported halitosis was independently associated with poorer OHIP-14 scores, together with other oral conditions such as xerostomia, dental caries, and dentine hypersensitivity [18]. Comparable findings have been reported in clinically assessed populations. In a Chinese study comparing 102 patients with halitosis and 102 controls, participants with halitosis had substantially higher OHIP-14 scores, and psychological discomfort and psychological disability were among the most frequently affected domains [19]. The importance of subjective symptoms is also supported by longitudinal cohort-derived evidence. Among 539 young adults from the 1982 Pelotas birth cohort, self-reported halitosis was independently associated with poorer OHIP-14 scores, and its association with OHRQoL was stronger than that observed for several clinically assessed oral conditions [20]. Interestingly, periodontitis also showed an indirect association with poorer OHRQoL through self-reported halitosis, suggesting that patients’ perception of malodor may represent an important pathway through which clinical oral disease affects daily life. These findings further indicate that halitosis can have a measurable impact on general OHRQoL, particularly through its psychological consequences. Similar associations have also been observed in younger populations; among 361 Nigerian adolescents, self-reported halitosis was associated with significantly poorer OHIP-14 scores [21], suggesting that the perceived psychosocial burden of malodor is evident across different age groups and cultural settings. These findings support an association between halitosis and general OHRQoL at the population level but do not necessarily imply that a generic OHRQoL instrument and a halitosis-specific measure should demonstrate strong individual-level correlations. Moreover, the recent Thai HALT validation found that T-HALT was moderately associated with self-perceived halitosis but was not significantly associated with objectively measured volatile sulfur compounds [3].

The distinction between perceived and clinically measured halitosis is particularly relevant when interpreting patient-reported quality-of-life outcomes. In a clinical study of 100 adults with objectively confirmed intra-oral halitosis, higher volatile sulfur compound levels were associated with poorer quality-of-life outcomes, particularly in social functioning, vitality, emotional well-being, and general health perception [22]. At the same time, systematic evidence on self-perceived halitosis indicates that psychological factors, including anxiety, self-esteem, body image, and interpretation of others’ reactions, can substantially influence the perceived burden of malodor [4].

The role of psychosocial factors is also evident among patients with objectively confirmed halitosis. In a study of 262 patients with genuine halitosis diagnosed by organoleptic assessment, more than one-fifth were classified as being at high risk of social anxiety disorder. Although oral malodor parameters and anxiety decreased after treatment, patients with high social-anxiety scores remained more likely to experience substantial anxiety after treatment [23]. This finding illustrates that the psychosocial burden associated with halitosis may persist partly independently of improvement in clinical malodor parameters.

In the present study, the absence of objective halitosis measurements limits our ability to distinguish the contribution of clinically measurable malodor from patients’ perceptions and psychosocial responses. The limited associations with OHIP-14 should therefore be interpreted as unresolved evidence concerning construct validity, rather than as confirmation of HALT-TR’s specificity. Moreover, although both questionnaire scores decreased at the group level, the lack of correlation between their individual change scores indicates that participants with larger improvements in HALT-TR did not consistently show larger improvements in OHIP-14. This distinction is important when considering the instruments for monitoring treatment outcomes: the observed group-level improvements do not establish that they provide interchangeable information about individual patients.

HALT-TR scores decreased from 38.33 ± 15.42 at baseline to 9.58 ± 14.13 one week after completion of dental treatment, with a mean reduction of 28.76 points and an SRM of 1.35. These findings demonstrate a large standardized short-term change in patient-reported halitosis-related quality of life. However, the magnitude of a pre–post change should be distinguished from evidence that an instrument validly measures change in its intended construct. Contemporary COSMIN guidance and consensus terminology treat responsiveness as a distinct measurement property requiring hypothesis-based evaluation [9,24]. The SRM should therefore be interpreted as a descriptive measure of standardized change rather than, by itself, definitive evidence of responsiveness or clinically meaningful improvement.

The improvement observed after periodontal and/or restorative treatment is also biologically and clinically plausible given the established association between periodontal disease and perceived oral malodor. In the 1982 Pelotas birth cohort, periodontitis was associated with a substantially increased likelihood of self-reported halitosis even after adjustment for relevant confounders [25]. Recent intervention evidence also provides context for the observed score changes. In a secondary analysis of a randomized controlled trial involving 24 patients with periodontitis, a 45-day strict oral hygiene intervention was associated with an approximately 11.9-point reduction in HALT scores in the intervention group [26]. This finding supports the possibility that patient-perceived halitosis-related burden may improve following oral care. However, direct comparison with the 28.76-point reduction observed in the present study is limited by differences in patient selection, intervention, follow-up timing, and study design. Our assessment occurred one week after completion of individually indicated periodontal and/or restorative treatment and did not include an untreated comparison group. The larger numerical reduction should therefore not be interpreted as evidence of superior treatment efficacy or greater responsiveness of HALT-TR.

Nevertheless, without an untreated comparison group or objective halitosis measurements, the observed improvement cannot be attributed exclusively to a reduction in oral malodor. Changes in other oral symptoms, treatment expectations, and natural variation may also have contributed. Furthermore, no patient-reported external anchor was available to estimate a minimal important change, and measurement error was not assessed. Consequently, the present results do not establish how much change represents a meaningful improvement for an individual patient or exceeds measurement error. Assessment one week after treatment completion also limits interpretation to short-term outcomes; the persistence of the observed improvement remains unknown.

The secondary analyses indicated that the reduction in HALT-TR scores was generally consistent across sex and educational-level groups, as reflected by the absence of significant interactions with time. Some associations were observed for age and marital status, including a significant marital status × time interaction and weak correlations between age and HALT-TR scores. These findings should be regarded as exploratory rather than as evidence of stable demographic differences in HALT-TR performance. In particular, some marital-status categories contained relatively few participants, limiting the reliability of subgroup comparisons. Previous research indicates that the relationship between halitosis and its psychosocial consequences can be influenced by individual and contextual factors, but the available evidence remains heterogeneous [1]. Larger and more demographically balanced samples are therefore needed before conclusions can be drawn regarding differential HALT-TR performance across sociodemographic groups.

The study has several strengths. The adaptation process included translation, review of semantic and conceptual equivalence, independent back-translation, and pilot testing in 30 individuals, with favorable pilot feedback and no subsequent changes to item wording. Evaluation in 200 adults included item-level analyses, dimensional assessment, comparison with a broader OHRQoL instrument, and repeated measurements following clinically indicated treatment. This combination provides information beyond a single internal consistency coefficient and identifies both promising findings and unresolved measurement properties. The standardized timing of the post-treatment assessment, one week after treatment completion, also provides a clearly defined context for interpreting short-term score changes.

Several limitations should be considered. First, recruitment from a single university dental center limits generalizability beyond treatment-seeking adults reporting halitosis. Most participants had university or postgraduate education, and the findings may not extend to populations with lower educational attainment or limited literacy. Small numbers in some sociodemographic categories also limit the interpretation of subgroup analyses. Furthermore, recruitment was based on halitosis complaints without organoleptic assessment or volatile sulfur compound measurement. The study therefore evaluates perceived halitosis-related burden rather than measurement performance in a population with objectively confirmed halitosis.

Second, important aspects of measurement quality remain unresolved. Although translation review and pilot testing addressed linguistic clarity and cultural appropriateness, the reported procedures do not establish comprehensive evidence of content validity or measurement equivalence across languages. Test–retest reliability and measurement error were not evaluated. Because the second administration followed treatment, when genuine change was possible, these repeated measurements cannot be used to establish temporal stability. The limited associations with OHIP-14 also leave convergent validity insufficiently supported; OHIP-14 was a related measure, not a gold standard for halitosis-specific quality of life. Pilot feedback was favorable, but structured cognitive interviews and formal item-level content-validity ratings were not documented.

Third, PCA and CFA were conducted in the same sample, and the proposed three-component model showed inadequate fit. The analyses therefore do not provide independent confirmation of the dimensional structure. Retaining the original 20-item total score preserved the original scoring approach but did not resolve its structural validity. The weak corrected item–total correlations for the first three items warrant further investigation of item interpretation and performance in an independent sample before decisions about item retention or removal are made. Although the additional ordinal common-factor analysis addressed the use of PCA and the eigenvalue >1 rule, it used the same baseline sample and did not resolve the weak definition of the third factor or provide independent validation. Future confirmatory work should use an independent sample and an estimator appropriate for ordered categorical responses [27].

Finally, participants received periodontal and/or restorative treatment according to individual clinical needs rather than a uniform intervention. The uncontrolled design cannot distinguish the contributions of different treatments or other influences on the observed changes. Follow-up was limited to one week after treatment completion, so the persistence of improvement is unknown. The absence of an external anchor for meaningful improvement and an estimate of measurement error also prevents individual score changes from being classified as clinically important or distinguishable from measurement noise. Future studies should prioritize independent structural validation, stable-condition repeat assessments, and longer-term follow-up with appropriate external measures to improve the interpretation of HALT-TR scores. The individual treatment categories and baseline clinical findings were not available for reporting, limiting assessment of treatment heterogeneity and preventing a clinical treatment-characteristics table.

## 5. Conclusions

This study provides preliminary evidence regarding the measurement performance of HALT-TR in adults with self-perceived halitosis. HALT-TR showed high internal consistency and a large standardized reduction in scores one week after completion of dental treatment. However, the proposed three-component structure was not supported, and associations with OHIP-14 provided limited evidence of convergent validity. The original 20-item total score was retained for analysis, but its structural validity and the clinical meaning of individual score changes remain unresolved. The current evidence does not support routine clinical use or the confirmed interpretation of the single total score. These findings provide a basis for further evaluation of HALT-TR, including independent structural validation, test–retest reliability, measurement error, and anchor-based interpretation of longitudinal changes.

## Figures and Tables

**Table 1:** Participant sociodemographic characteristics.

Variable	Value
Participants, *n*	200
Age	
Age, years, mean ± SD	40.20 ± 15.52
Age, median	37
Age range, years	19–87
Gender	
Female, *n* (%)	129 (64.5)
Male, *n* (%)	71 (35.5)
Education	
High School, *n* (%)	17 (8.5)
University, *n* (%)	118 (59.0)
Postgraduate, *n* (%)	65 (32.5)
Marital status	
Single, *n* (%)	70 (35.0)
Divorced, *n* (%)	9 (4.5)
Widowed, *n* (%)	4 (2.0)
Married, *n* (%)	117 (58.5)

**Table 2:** Pretreatment item analysis and internal consistency of HALT-TR.

HALT Item (Brief Content)	Corrected Item–Total Correlation	Cronbach’s α If Item Deleted
1. Mouth breathing frequency	−0.004	0.925
2. Throat infection frequency	0.088	0.922
3. Sinusitis frequency	0.242	0.921
4. Concern about bad breath	0.432	0.917
5. Unhappiness or tension	0.589	0.913
6. Chewing difficulty or food avoidance	0.647	0.912
7. Taste alteration	0.742	0.910
8. Speech difficulty or covering mouth	0.762	0.910
9. Perceived effect on appearance	0.747	0.909
10. Feeling depressed	0.803	0.909
11. Difficulty concentrating	0.783	0.909
12. Embarrassment	0.657	0.912
13. Time spent learning odor remedies	0.371	0.919
14. Keeping distance when speaking	0.629	0.913
15. Avoiding outings or social gatherings	0.706	0.911
16. Communication difficulty	0.776	0.909
17. Others mentioning bad breath	0.535	0.915
18. Financial loss	0.588	0.914
19. Social or personal relationship loss	0.594	0.913
20. Reduced quality of life	0.638	0.912

Overall Cronbach’s α = 0.918; *n* = 200.

**Table 3:** (**A**) Exploratory common-factor loadings from the polychoric correlation matrix (Varimax rotation; *n* = 200). (**B**) Confirmatory factor analysis fit indices for the PCA-derived HALT-TR model.

(**A**)											
**HALT Item**			**Factor 1**			**Factor 2**			**Factor 3**		
1			−0.112			0.072			0.317		
2			0.011			0.045			0.988		
3			0.243			0.097			0.518		
4			−0.077			0.822			0.155		
5			0.073			0.902			0.105		
6			0.571			0.408			0.100		
7			0.707			0.412			0.081		
8			0.560			0.588			0.072		
9			0.484			0.660			0.012		
10			0.650			0.586			−0.041		
11			0.652			0.554			−0.036		
12			0.218			0.820			−0.033		
13			−0.015			0.622			0.067		
14			0.304			0.720			−0.167		
15			0.690			0.419			−0.096		
16			0.758			0.419			−0.020		
17			0.820			−0.006			0.165		
18			0.963			−0.013			0.046		
19			0.954			−0.004			−0.022		
20			0.728			0.236			−0.071		
(**B**)											
**Model**		**χ^2^/df**		**RMSEA**		**SRMR**		**CFI**		**GFI**	
Three-component model		7.22		0.18		0.13		0.88		0.62	

**Table 4:** Correlations between HALT-TR and OHIP-14 scores.

Comparison	Spearman’s ρ (95% CI)	*p*-Value
Pretreatment HALT-TR vs. pretreatment OHIP-14	−0.080 (−0.218 to 0.059)	0.259
Post-treatment HALT-TR vs. post-treatment OHIP-14	0.266 (0.124 to 0.397)	<0.001
ΔHALT vs. ΔOHIP-14	−0.091 (−0.231 to 0.048)	0.200

**Table 5:** Pretreatment and post-treatment changes in HALT-TR and OHIP-14 scores.

Scale	Pretreatment Mean ± SD Median [Q1–Q3]	Post-Treatment Mean ± SD Median [Q1–Q3]	Change Mean ± SD; 95% CI Median [Q1–Q3]	SRM	Wilcoxon W; *p*
HALT-TR	38.33 ± 15.42 40.5 [28–48]	9.58 ± 14.13 3 [0–13.25]	28.76 ± 21.32 25.78–31.73 33 [18.5–42]	1.35	803 <0.0001
OHIP-14	26.06 ± 9.56 28 [17–31]	5.98 ± 6.84 3.5 [0–11]	20.08 ± 8.13 18.95–21.21 20 [14–27]	2.47	4 <0.0001

Abbreviations: CI, confidence interval; SD, standard deviation; SRM, standardized response mean.

## Data Availability

The data presented in this study are available from the corresponding author upon reasonable request. The data are not publicly available due to ethical and privacy considerations related to participant confidentiality and the conditions of the ethical approval.

## Abbreviations

ATS, ANOVA	type statistic
CFA	confirmatory factor analysis
CFI	comparative fit index
CI	confidence interval
COSMIN	COnsensus-based Standards for the selection of health Measurement INstruments
GFI	goodness-of-fit index
HALT	Halitosis Associated Life-Quality Test
HALT-TR	Turkish version of the Halitosis Associated Life-Quality Test
ICC	intraclass correlation coefficient
IQR	interquartile range
KMO	Kaiser–Meyer–Olkin
OHIP	Oral Health Impact Profile
OHIP-14	Oral Health Impact Profile-14
OHRQoL	oral health-related quality of life
OIDP	Oral Impacts on Daily Performance
PCA	principal component analysis
PROM	patient-reported outcome measure
RMSEA	root mean square error of approximation
SD	standard deviation
SF-36	36-Item Short Form Health Survey
SRM	standardized response mean
SRMR	standardized root mean square residual
VSC	volatile sulfur compound
